# Supplementary material for: TrimNN: Characterizing cellular community motifs for studying multicellular topological organization in complex tissues
Source: Res Sq. 2025 Jan 17:rs.3.rs-5584635. Preprint. [Version 1] doi: 10.21203/rs.3.rs-5584635/v1 (PMC11774463; doi:10.21203/rs.3.rs-5584635/v1)
Supplement: Supplement 1 [file NIHPPrs5584635v1-supplement-1.pdf]

Supplementary Files

This is a list of supplementary files associated with this preprint. Click to download.

- SupplementaryNotes20241204.pdf
- SupplementaryData20241202.xlsx
- SupplementaryFigure20241204.pdf
